# Psychometric Properties of the Cyprus Lexical List in the Greek Language for Infants and Preschool Children and Preliminary Results

**DOI:** 10.3389/fpsyg.2022.846249

**Published:** 2022-07-04

**Authors:** Meropi Helidoni, Areti Okalidou, Alexandra Economou, Elli Spyropoulou, Kakia Petinou

**Affiliations:** ^1^General University Hospital of Heraklion, Heraklion, Greece; ^2^Department of Educational and Social Policy, University of Macedonia, Thessaloniki, Greece; ^3^Department of Psychology, School of Philosophy, National and Kapodistrian University of Athens, Athens, Greece; ^4^Department of Psychology, University of Crete, Rethymno, Greece; ^5^Department of Rehabilitation Sciences, Cyprus University of Technology, Limassol, Cyprus

**Keywords:** receptive vocabulary, expressive vocabulary, adapted Cyprus Greek Lexical List, demographic variables, psychometric properties

## Abstract

The aim of this study was to evaluate the psychometric properties of the adapted Cyprus Greek Lexical List a-CYLEX (GR) in a sample of 194 Greek toddlers from the island of Crete with Standard Modern Greek (SMG) as their primary language. The a-CYLEX (GR) is a parental report checklist for assessing the receptive and expressive vocabulary skills of children aged 12 months to 3:6 years. Concurrent validity of the instrument was tested via correlations with the adapted Greek version of the Receptive One-Word Picture Vocabulary Test-II (ROWPVT-II), which was administered to 124 SMG-speaking children between the ages of 2 and 3:6 years. Test–retest reliability was tested by administering the instrument two times within a 2-week interval to 59 parents (30.41% of the total sample). Statistical analyses provided strong evidence for the high internal consistency and test–retest reliability of the a-CYLEX (GR). The role of the demographic variables in vocabulary performance and the frequency of each a-CYLEX (GR) word category by age were also investigated. In conclusion, the a-CYLEX (GR) is a parental report checklist that can be used by clinicians who are interested in assessing receptive and expressive vocabulary of children during toddlerhood.

## Introduction

Typically developing children follow certain language milestones in order to acquire adult like language skills, despite variability in developmental trajectories (Berko-Gleason and Ratner, 2016). Meaningful words emerge at around the age of 12 months with a remarkable acceleration of early productive words at the age of 20 months (Stoel-Gammon, 1989; Bates et al., 1994). Rescorla (1989) and Rescorla and Dale (2013) reported that by the age of 2 years, typically developing English-speaking children presented with a mean productive vocabulary of at least 50 meaningful words as well as the emergence of two and three-word phrases. Between the ages of 18–20 months, youngsters present with a “critical mass” of productive words which form the bases for phonological, semantic, and morphosyntactic development (Marchman and Bates, 1994). Important interactions have been identified between the development of phonology and lexicon. Phonological ability affects lexical acquisition and the lexicon influences phonological knowledge (Stoel-Gammon, 2011). Petinou et al. (2021) investigated the existence of interconnectedness between developing linguistic subsystems in 31 typically developing Greek-speaking toddlers at 28 and 36 months and found significant positive relationships among all language skills.

Along these lines, a remarkable vocabulary acceleration in the form of a vocabulary “spurt” occurs right after children reach the 50-word production milestone (Hoff, 2005; Berko-Gleason and Ratner, 2016). On the other hand, Mervis and Bertrand (1995) maintain that the vocabulary spurt might occur at a mean vocabulary size of 112 words in English-speaking toddlers. It has been argued that the existence of a vocabulary “spurt” should be re-examined in order to accommodate the variability in terms of expressive language acceleration observed at different ages for different children (Ganger and Brent, 2004). Nevertheless, expressive vocabulary development constitutes a linguistic component with significant research and clinical implications. Thus, receptive and expressive vocabulary form the impetus and platform for a cross-language project on parentally reported information regarding the child’s vocabulary development (Fenson et al., 1993).

D’Odorico et al. (2001) investigated developmental language patterns on 42 Italian-speaking children and found that toddlers who exhibited a vocabulary spurt at an earlier chronological age, progressed faster in lexical gains, regardless of developmental variability. The number of words produced by children appears to play a significant role in determining early expressive language delays (Stoel-Gammon, 2010; Rescorla and Dale, 2013).

Examining and documenting lexical development remains crucial as this skill predicts later language and academic challenges (Rescorla and Dale, 2013). The mapping of semantic skills as a function of age provides information for both typically developing toddlers as well as toddlers with protracted linguistic onset. A language disorder is typically observed when a child’s language skills deviate significantly from what is typically expected for his/her chronological age (Leonard, 2014). According to the fifth edition of the Diagnostic and Statistical Manual of Mental Disorders (DSM - V), language disorder refers to persistent difficulties in the comprehension or production of spoken language, written language, sign language or other forms of language where language skills are below age expectations (American Psychiatric Association, 2013).

Rescorla (1989) reported that at 24 months, 10% of middle-class toddlers did not use a 50-word vocabulary and did not combine two words into phrases, thus suggesting that late-talking is quite common at this age. Although 70% of the children with language delay may reach other children of the same age when they are 3 years old, in 30% of the children the language delay remains (Rescorla and Schwartz, 1990; Paul et al., 1991). Other studies have also shown that many toddlers with early delay appear to catch up with typically developing peers by the time they reach the age of 3 years (Paul and Jennings, 1992; Petinou and Okalidou, 2006; Rescorla, 2013; Petinou and Spanoudis, 2014). Children at 36 months whose non-verbal cognitive abilities are within the normal range and expressive language delay persists might develop a developmental language disorder which may persist in the school-age years (Desmarais et al., 2008; Petinou et al., 2011).

Numerous studies suggest that early linguistic challenges in the form of a language delay during the preschool years forms a significant risk factor for chronic language impairment with associated academic failure and learning disabilities (Scarborough and Dobrich, 1990; Nash and Donaldson, 2005; Wise et al., 2007; Desmarais et al., 2008; Rescorla, 2009; Petinou et al., 2011; Bleses et al., 2016). Late talkers might be at risk of developing a language disorder compared to typically developing children (Rescorla and Schwartz, 1990; Rescorla et al., 2000b; Parizi et al., 2013). Although many late-talking children exhibit a significant improvement in their vocabulary development as they grow older, they show persistent language challenges in the areas of phonological intelligibility, morphological skills, narrative skills and sentence structure complexity (Paul and Alforde, 1993; Mirak and Rescorla, 1998; Rescorla and Turner, 2015). These challenges are accompanied by a pattern of delayed grammar use, a restricted mean length of utterance as well as immature phonetic inventories (Rescorla et al., 2000a; Petinou et al., 2011). More specifically, Rescorla and Ratner (1996) found that 30, 24-month-old toddlers with specific expressive language delay had a restricted consonantal inventory along with immature syllable structure use. Such observations were corroborated by experimental data reported for Cypriot-Greek (CG) with late onset of expressive language. Petinou and Okalidou (2006) investigated the speech patterns in seven CG late talkers at 30, 33, and 36 months and found that they presented poorer phonetic inventories compared to an age-matched control group. Hammer et al. (2017) investigated whether children who were late talkers at 24 months old would continue to have low vocabulary at 48 months old and whether school readiness would be affected at 60 months old. Interestingly, they found that one fourth of these children would continue to present with low vocabulary at 48 months old and at 60 months these children would be at risk for reduced school readiness due to low reading and low math scores and behavior problems. Furthermore, Grossheinrich et al., 2019) reported that 39 German-speaking children who had been identified as late talkers at the age of 24 months old presented significant differences in language and literacy skills in third-grade in comparison to 39 typically developing children who also attended the third grade.

Notably, the examination of developmental language patterns in Greek-speaking children remains quite sparse (Tsimpli, 2001; Papaeliou and Rescorla, 2011). Nevertheless, a number of data-driven examinations on expressive and receptive language provide a preliminary database that can be used as a benchmark for further assessments. Okalidou and Kampanaros (2001) conducted a study in which 1113 Greek children attending kindergarten were assessed by their teachers using an adapted Communication Checklist for Preschool Teachers (CCPT), in order to identify communication problems. Based on teacher estimates, approximately 14.4% – 18.7 % of children displayed communication impairments. A higher prevalence rate of communication problems was found for boys than for girls. Also, Karousou and Petrogiannis (2014) assessed 1391 Greek – speaking infants and toddlers aged 7–30 months with a new parental report instrument (Communication Development Report), (Karousou and Petrogiannis, 2014), which measures communication and language development. They reported that language comprehension of words begins to takes place before the age of 8 months and children’s first words appear between 12 and 13 months.

Overall, these findings support the existence of certain developmental milestones for the acquisition of language, with frequent inter-individual variability which is considered a variation of the typical course of developmental language trajectories. Nevertheless, as mentioned earlier, the language delay may persist after children start school and might lead to academic and behavioral problems. Therefore, a careful investigation of children’s language skills is paramount.

An additional parameter regarding vocabulary development pertains to semantic profiling and variability reported across ages and across languages (Crystal, 1997; Maier et al., 2016). Benedict (1979) reported that from the beginning of language development children learn different classes of words such as nominals, action words, modifiers, and personal-social words. However, the most outstanding categories are nominals and action words. Studies in English-speaking and Italian-speaking children have indicated that children first acquire nouns, whereas action words emerge later (Bates et al., 1994; Caselli et al., 1995; Bergelson and Swingley, 2013, 2016). In the study by D’Odorico et al. (2001) in the first 50-word stage the most frequent vocabulary items used by the children were onomatopoeic words and names of people and at the 100 and 200 word-stages the most prominent lexical items used by the children were nouns. Similar findings were obtained for Greek-speaking children by Papaeliou and Rescorla (2011), who examined vocabulary size and lexical profiling in 273 Greek-speaking children aged 1 year and 6 months to 2 years and 11 months using the Greek adaptation of the Language Development Survey (Rescorla, 1989) and found that nouns were the most prominent category referring mostly to the semantic category of “people.” According to Stephany (1997) verbs constitute the larger percentage of words Greek children hear. Papaeliou and Rescorla (2011) mention that verbs are more often the first element in sentences children hear in the Greek Language in comparison to the English Language. Benedict (1979) reported that action words constituted a considerably larger proportion of the early comprehension vocabulary of children, while nominals were more prominent in their productive vocabulary. However, it should be taken under consideration that for comprehension action words might be easier assessed in comparison to objects words (Benedict, 1979).

Kern (2007) investigated the development of early lexicon in 548 French infants and found that in infants aged from 8 to 16 months nouns were predominant in their receptive and expressive vocabulary with action words increasing as the lexicon increased. However, primary noun acquisition in early vocabulary has also been questioned by studies in other languages (Choi and Gopnik, 1995 in Korean; Tardif, 1996 in Mandarin). The investigators studied the pattern of vocabulary development in nine Korean-speaking and 10 Mandarin-speaking children and found that verbs were acquired first in children’s languages.

Numerous studies have reported that gender, parental education, and kindergarten attendance have an effect on children’s language development (Huttenlocher et al., 1991; Arriaga et al., 1998; Bornstein and Haynes, 1998; Feldman et al., 2000; D’Odorico et al., 2001; Bauer et al., 2002; Bavin et al., 2008; Umek et al., 2008; Eriksson et al., 2012; Hammer et al., 2017). Bouchard et al. (2009) used the Quebec French version of the MacArthur Communicative Developmental Inventories (Trudeau et al., 1999) to investigate gender differences in language development in French Canadian children between 8 and 30 months of age. They found that between 8- and 16-months girls understood more sentences and produced more words than boys, whereas between 16- and 30-months girls produced more words than boys, their utterances consisted of more grammatical forms, and their syntax skills were more advanced. Interestingly, they found that the difference in language competence of girls in comparison to boys became less evident after 28 months, where boys seemed to catch up to girls. In addition, Roberts et al. (1999) reported that children from more stimulating environments had larger vocabularies and used longer utterances. Furthermore, girls in the same study showed a better performance in language skills compared to boys. Hoff (2003) found that children from higher socioeconomic families had better vocabulary development in comparison to children from mid socioeconomic status because mothers used longer utterances and a richer vocabulary in everyday interactions. Ansari and Winsler (2011) investigated 6929 4-year-old Latino children who did or did not attend nursery school and found that children who spent more time at a nursery improved with time in their cognitive, linguistic, and social skills, while those who were kept in the home environment did not show improvement in these areas. Furthermore, Hansen and Hawkes (2009) investigated expressive vocabulary, cognitive skills, and problem behavior of the children of 1737 families and found that those who attended formal group care from 9 months had a better expressive vocabulary, positive cognitive outcomes and better school readiness at 3 years of age, but there was no association between formal group care and problem behavior. In conclusion, researchers should consider the aforementioned variables when investigating lexical production, comprehension and diversity.

Many studies have used the method of parental report to measure vocabulary development. A parental vocabulary list provides significant information related to children’s receptive and expressive vocabulary status at certain ages that are critical to language development. Such a list is easy to complete and inexpensive. Two parental vocabulary checklists that are widely used are the MacArthur Communicative Development Inventories (CDI) (Fenson et al., 1993) and the Language Development Survey (LDS) (Rescorla, 1989). Both the CDI (Bates et al., 1994; D’Odorico et al., 2001; Heilmann et al., 2005; Kern, 2007; Wehberg et al., 2007; Stolt et al., 2008) and the LDS (Rescorla and Alley, 2001; Rescorla and Achenbach, 2002) have been used by various studies to measure vocabulary development in different countries. A more recent parental report instrument for the early screening of communication and language development in Greek–speaking infants and toddlers is the Communication Development Report, which was found to be valid and reliable (Karousou and Petrogiannis, 2014).

The Cyprus Lexical List (CYLEX) was originally developed by Petinou et al. (1999) in Cyprus. CYLEX is a parental report vocabulary checklist based on the structural principles of the MacArthur Communicative Development Inventory (Fenson et al., 1993). CYLEX measures receptive and expressive vocabulary by requesting the parent to check whether the child understands and/or produces certain words from different semantic categories.

The CYLEX parental report checklist has been used in several studies in Greece and Cyprus (Petinou et al., 2011; Parizi et al., 2013; Oktapoti et al., 2016; Salagoudi, 2019). In Cyprus, Petinou et al. (2011) used CYLEX combined with other assessments in order to investigate the language skills of Cypriot-Greek speaking toddlers with specific language delay. In Greece, the a-CYLEX, which is an adaptation of CYLEX into standard Greek, has been used by Parizi (2012) who examined vocabulary development in 200 typically developing Greek children from 6 months to 3 years and 6 months, by Chachoudi (2012), who investigated the development of semantic categories in 200 typically developing children in the same age range, and by Salagoudi (2019) who investigated the semantic development and diversity in 50 typically developing children aged 1.5–4 years old.

Finally, Oktapoti et al. (2016) measured the vocabulary development of 13 deaf Greek speaking children with cochlear implants aged between 21 and 71 months and compared these data with data previously collected from typically developing hearing Greek-speaking children in three age groups, i.e., 24–26, 28–30, and 36–38 months. The results indicated that the vocabulary skills of the implanted children with a mean post-implant age of 20 months were similar to those of typically developing hearing Greek-speaking children in the age group 24–26 months. The a–CYLEX was found to be a reliable and useful tool for exploring receptive and expressive vocabulary development with this clinical population.

A wealth of significant conclusions from the above studies, both in typically developing and in clinical populations, has given rise to the need to continue the process of standardization of the a-CYLEX in Greek by using a sample from the island of Crete. More word changes were made in order to better adapt a-CYLEX into standard Greek and the name of the parental checklist was changed to a-CYLEX (GR).

The aim of the present study was to measure the psychometric properties of the new Greek adapted version of the a-CYLEX parental checklist in the island of Crete. More specifically the objectives of this study were as follows:

(1) To determine the test–retest reliability, internal consistency, and concurrent validity of the receptive vocabulary of a-CYLEX (GR) inventory which was conducted in the island of Crete.
(2) To present the frequency of each a-CYLEX (GR) word category of receptive and expressive vocabulary by age and gender, and the differences of word categories by gender.
(3) To assess the influence of several demographic factors on children’s vocabulary development (age, gender, kindergarten attendance, educational status of parents).

## Materials and Methods

### Participants

The sample of the present study consisted of 194 toddlers [mean age = 28.00 months, *SD* (9.07); 54.1% girls]. The majority of participants came from urban areas in Crete (Heraklion, Rethimno, Agios Nikolaos), and the remaining 7.7% (*n* = 14) from a rural area of Heraklion district (Venerato, Agios Mironas, Fodele, Viannos, Agia Varvara, Augeniki). Regarding the educational status of parents who completed the a-CYLEX (GR), 1% of the parents completed primary school, 37.6% attained secondary school, 47.4% completed a Bachelor’s degree, and 14.4% a Master’s degree respectively. Of the above sample, 74.7% (*n* = 145) of the children attended public (65%; *n* = 127) or private (12.4%; *n* = 18) kindergarten. Based on parental reports all the children who took part in the study had typical motor and cognitive development, although for 6.2% of the children (*n* = 12), parents expressed concern about their children’s expressive language output. Additionally, 2.6% (*n* = 5) of the children had already visited a health specialist for an assessment without receiving an official diagnosis. For research purposes the sample was divided into 7 age groups with an interval of 6 months (0:6–0:11, 1:0–1:5, 1:6–1:11, 2:0–2:5, 2:6–2:11, 3:0–3:5, and 3:6). Detailed demographic information per age group and information regarding the parents who completed the a-CYLEX (GR) is presented in Table 1. Although the CYLEX was originally intended for children aged 12 months to 3:6 years, following the studies of Chachoudi (2012) and Parizi et al. (2013), in the present study the a-CYLEX (GR) was used for infants from the age of 6 months.

**TABLE 1:** Demographics of participants in a-CYLEX (GR) per age groups.

Age Groups (*n*)	0:6–0:11 (*n* = 8)	1:0–1:5 (*n* = 17)	1:6–1:11 (*n* = 14)	2:0–2:5 (*n* = 36)	2:6–2:11 (*n* = 50)	3:0 – 3:5 (*n* = 64)	3:6 (*n* = 5)
**Age in Months**							
M ean (*SD*)	8.25 (*2.00*)	14.35 (*2.00*)	21 (2.50)	26 (*1.70*)	31.7 (*3.00*)	38.1 (*1.90*)	42.0 (*0.00*)
**Gender**							
Boys (%)	25%	70.6%	78.6%	39%	36%	45.3%	60%
**Siblings**							
yes (%)	87.5%	47.1%	64.3%	55.6%	54%	76.6%	60%
**Status Residence**							
City (%)	87.5%	100%	100%	97.2%	94%	87.5%	80%
**Kindergarten**							
Attendance (%)	100%	17.6%	80%	96.2%	97.5%	15.5%	40%
Public (%)	12.5%	17.6%	80%	96.2%	97.5%	15.5%	40%
**Hearing problems (%)**	0%	0%	0%	0%	0%	0%	0%
Glue Ear (%)	28.6%	6.7%	7.7%	2.9%	13%	18.6%	0% 25%
**Participants Parent’s gender**							
Female (%)	100%	94.1%	92.9%	88.9%	94%	93.8%	80%
Parent’s age							
M ean (*SD*)	35.80 (*2.60*)	32.50 (*3.60*)	31.40 (*4.90*)	34.60 (*4.40*)	35.40 (*5.50*)	34.90 (*4.60*)	33.80 (*3.80*)
**Parent’s educational level (%)** Primary School	–	–	–	–	–	1.60%	–
Secondary School	50.00%	17.60%	21.40%	36.18%	44.00%	39.10%	60.00%
Bachelor	25.00%	70.60%	57.10%	44.40%	40.00%	51.60%	20.00%
Master	25.00%	11.80%	21.40%	19.40%	16.00%	7.80%	20.00%

The study was approved by the Bioethical Committee of the University of Macedonia. All participants were informed in detail about the aims of the study and were guaranteed the anonymity and confidentiality of the data. Written consent was a prerequisite for participation. Participation in the study was voluntary and no compensation was given.

### Measures

An adaptation of the a-CYLEX (Parizi et al., 2013), the a-CYLEX (GR) was used in this study. The parental checklist a-CYLEX (GR) assesses receptive and expressive language skills. The same words are included in both the receptive and expressive language sections.

Parizi et al. (2013) adapted the original CYLEX in the Standard Greek Language. Sixty-four words were translated from Cypriot-Greek to Standard Greek. The a-CYLEX used by Chachoudi (2012); Parizi (2012), and Salagoudi (2019) consisted of 611 items as in the original CYLEX most frequently found in Cypriot-speaking children’s speech. They are separated into the following 18 semantic categories: baby words, animal sounds, animal names, food/drink, body parts, actions, places (outside things), household objects, rooms, personal items, people, vehicles, clothes, concepts, adjectives, tools, toys and other words. In the a-CYLEX there are four more sections where parents can complete names, numbers, and other words (not provided by the list) that their child understands and/or says and sentences that their child understands/says. Several sentence examples that the child says can be provided by the parents in the section. The aforementioned sections were not taken into consideration in the present study. The a-CYLEX takes about 30 min to complete.

Taking into consideration this dialectal variation, and in order to better adapt a-CYLEX into standard Greek, the authors made the following changes to the a-CYLEX (Parizi et al., 2013). In the “food/drink category” one word alternative was added to the already used word “glykisma” that Greek children might better understand in Southern Greece, i.e., “glykisma” “glykisma/gliko” (sweet) and “mastixa,” “tsixla” (gum) which were separate, were combined into one “mastixa/tsixla.” Two words were omitted from the “food/drink category” because they are brand names (Coca-cola and Nescafe) and two were omitted because they are foreign words for which there are Greek equivalents (chips, hamburger). In the “body parts category,” the two items “pisinos,” “popos” which mean buttocks were combined into one item “pisinos/popos.” In the “actions category” the word “siopi” (silence) was moved to the category “other words.” In the same category, the verb “tsibao” was added to the word “tsibo” as it contains an alternative morphological suffix, namely “tsibao/tsibo” (nibble or pinch). In the category “places” the word “Goodies” was omitted as it refers to a company and the word “farm” because it was already included in the “toys category.” Furthermore, from the “household objects category” the words “labater” (lamp), “camera” (camera), “caseta” (tape) were omitted because they are not frequently used by children. The word “ftiari” (spade) from the “household objects category” was moved to the “toys category.” In the “people category” the words “Theoulis” (God), “ktistis” (builder), “Panagitsa” (Virgin Mary) and “Xristoulis” (little Christ) were omitted in order to eliminate cultural/religious bias. Finally, in the “toys category” the words “bouloukos” (fatty), “penna” (fountain-pen) and “troxos” (wheel) were omitted because they are not frequently used by children (see Table 2). The a-CYLEX (Greek version – GR) used in this study consists of 594 words also separated in the same 18 semantic categories.

**TABLE 2:** Word changes in word categories between a-CYLEX and a-CYLEX(GR).

Word categories	Words a-CYLEX	Words a-CYLEX(GR)
“Food/Drink category”	glykisma (sweet)	glykisma/gliko (another word added)
	mastixa (gum) tsixla(gum)	combined – “mastixa/tsixla”
	coca-cola	omitted (brand name)
	nescafe	omitted (brand name)
	chips	omitted (foreign word)
	hamburger	omitted (foreign word)
“Body parts category”	pisinos (buttocks) popos (buttocks)	combined – “pisinos/popos”
“Actions category”	siopi (silence)	moved to the “other words category”
	tsibo (nibble or pinch)	tsibao/tsibo
“Places category”	Goodies	omitted (refers to a food company)
	farm	omitted (already included in the “toys category”)
“Household objects category”	labater (lamp)	omitted (not frequently used by children)
	camera	omitted (not frequently used by children)
	caseta (tape)	omitted (not frequently used by children)
	ftiari (spade)	moved to the “toys category”
“People category”	Theoulis (God)	omitted (to eliminate cultural/religious bias)
	Ktistis (builder)	omitted (to eliminate cultural/religious bias)
	Panagitsa (Virgin Mary)	omitted (to eliminate cultural/religious bias)
	Xristoulis (Little Christ)	omitted (to eliminate cultural/religious bias)
“Toys category”	bouloukos (fatty)	omitted (not frequently used by children)
	penna (fountain-pen)	omitted (not frequently used by children)
	troxos (wheel)	omitted (not frequently used by children)

The Receptive One-Word Picture Vocabulary Test-II (ROWPVT-II; Brownell, 2000) was employed in the present study as adapted and normed in the Greek Language by Okalidou et al. (2011). The ROWPVT-II assesses the receptive vocabulary of children aged from 2:0 to 5:11 years. It consists of 170 colored items. The examiner shows to the child a set of four pictures and the child is requested to point to the picture that the examiner asks for. It takes about 10–15 min to complete.

### Procedure

Data collection took place from 06/06/2017 until 23/06/2018. The a-CYLEX (GR) questionnaires were given to all the parents by the first author. The a-CYLEX (GR) checklist was accompanied by a letter that described the purpose of the study, a consent form and a questionnaire which requested demographic information. Seventy-four parents completed the questionnaires in the author’s private practice facility and during the time of completion of the questionnaires the first author administered the ROWPVT-II to the children aged 2:0–3:6 years. For the rest of the sample (120 participants) the author visited six public kindergartens after permission was provided by the appropriate authority of the Heraklion prefecture (Municipal Organization of Preschool Education). After the a-CYLEX (GR) checklists were collected, the author visited the kindergartens and administered the ROWPVT-II to the children aged 2:0 to 3:6 years, within a 3-month period. The author resolved queries the parents had regarding the completion of the a–CYLEX (GR) checklist in the private practice facility and at the kindergartens.

The a–CYLEX (GR) was completed twice by 59 parents (30.41% of the total sample) with a 2-week period between each administration for reliability purposes. An interval period of 2 weeks was decided upon because no substantial change was expected to take place in the children’s vocabulary development within that period and because the parents would probably not remember in detail what they had answered the first time. When the parents completed the a–CYLEX (GR) checklist for the second time, they had no access to the first a–CYLEX (GR) checklist they had completed the first time.

## Results

The Statistical Package for the Social Sciences, Version 25 (IBM Corp., 2017) was used for all statistical analyses. The psychometric parameters that were tested were test–retest reliability, internal consistency, and concurrent validity of a–CYLEX (GR).

### Reliability of the a–CYLEX (GR)

Test–retest reliability of the a–CYLEX (GR) was assessed by re-administering the instrument to 59 parents (30.41% of the total sample) 2 weeks after the initial assessment. The values between the two assessments are shown in the Appendix. Pearson *r* correlation coefficients were significantly high for both language skills, receptive vocabulary, *r* = 0.99, *p* < 0.001 and expressive vocabulary, *r* = 0.99, *p* < 0.001.

### Internal Consistency of the a-CYLEX (GR)

Internal consistency of the a–CYLEX (GR) was assessed with Cronbach’s α for the total score and for each semantic subscale separately a-CYLEX (GR), α = 0.98; Receptive Vocabulary, α = 0.96; Expressive Vocabulary, α = 0.98). Intercorrelations among a-CYLEX’s (GR) word categories using Pearson *r* were found to be statistically significant (*p* < 0.01) and ranged for the Receptive Vocabulary from 0.43 to 0.94, and for the Expressive Vocabulary from 0.41 to 0.97. In general, the smallest intercorrelations were found between baby words and other word categories for both Receptive (e.g., *r*_*babywords–actions*_ = 0.43) and Expressive Vocabulary (e.g., *r*_*babywords– tools*_ = 0.47). Correlation coefficients between Receptive and Expressive word categories in a-CYLEX (GR) and the total score on a-CYLEX (GR) were also significant (*p* < 0.01) and ranged from 0.50 to 0.97. Similarly, the smallest correlations were found between baby words for both Receptive and Expressive Vocabulary and the total score on a-CYLEX (GR). Thus, the internal consistency of the a–CYLEX (GR) checklist was supported (see Table 3).

**TABLE 3:** Intercorrelations between the word categories in a-CYLEX (GR) and the total score in a–CYLEX (GR).

Word Categories	1	2	3	4	5	6	7	8	9	10	11	12	13	14	15	16	17	18	19
(1) Baby Words	–	0.56**	0.47**	0.50**	0.47**	0.43**	0.45**	0.45**	0.41**	0.50**	0.45**	0.43**	0.43**	0.38**	0.38**	0.45**	0.43**	0.51**	0.50**
(2) Animal Sounds	0.59**	–	0.77**	0.76**	0.75**	0.72**	0.72**	0.70**	0.63**	0.75**	0.71**	0.69**	0.69**	0.66**	0.52**	0.72**	0.70**	0.71**	0.76**
(3) Animal Names	0.57**	0.77**	–	0.94**	0.93**	0.93**	0.93**	0.92**	0.85**	0.93**	0.93**	0.89**	0.91**	0.88**	0.73**	0.92**	0.91**	0.86**	0.95**
(4) Food/Drinks	0.59**	0.71**	0.87**	–	0.96**	0.96**	0.93**	0.93**	0.84**	0.94**	0.92**	0.87**	0.92**	0.88**	0.72**	0.91**	0.90**	0.90**	0.96**
(5) Body	0.61**	0.76**	0.86**	0.94**	–	0.95**	0.91**	0.92**	0.82**	0.93**	0.91**	0.86**	0.90**	0.87**	0.70**	0.90**	0.88**	0.89**	0.95**
(6) Actions	0.57**	0.73**	0.84**	0.92**	0.93**	–	0.96**	0.96**	0.88**	0.95**	0.95**	0.90**	0.96**	0.92**	0.76**	0.94**	0.93**	0.90**	0.98**
(7) Places	0.54**	0.63**	0.84**	0.89**	0.84**	0.89**	–	0.97**	0.93**	0.95**	0.97**	0.94**	0.96**	0.94**	0.83**	0.97**	0.95**	0.89**	0.98**
(8) House	0.59**	0.70**	0.83**	0.93**	0.91**	0.93**	0.92**	–	0.92**	0.96**	0.96**	0.93**	0.95**	0.92**	0.80**	0.96**	0.94**	0.88**	0.98**
(9) Rooms	0.53**	0.59**	0.76**	0.81**	0.75**	0.80**	0.88**	0.86**	–	0.89**	0.92**	0.91**	0.90**	0.91**	0.87**	0.93**	0.91**	0.81**	0.92**
(10) Personal	0.60**	0.72**	0.86**	0.92**	0.91**	0.92**	0.89**	0.93**	0.81**	–	0.95**	0.90**	0.94**	0.91**	0.77**	0.96**	0.94**	0.90**	0.98**
(11) People	0.54**	0.65**	0.84**	0.85**	0.81**	0.84**	0.92**	0.88**	0.84**	0.87**	–	0.93**	0.96**	0.95**	0.82**	0.96**	0.95**	0.91**	0.98**
(12) Vehicles	0.51**	0.58**	0.78**	0.79**	0.76**	0.80**	0.87**	0.84**	0.82**	0.77**	0.85**	–	0.92**	0.91**	0.86**	0.93**	0.94**	0.84**	0.94**
(13) Concepts	0.55**	0.65**	0.81**	0.86**	0.83**	0.90**	0.92**	0.88**	0.84**	0.86**	0.91**	0.83**	–	0.96**	0.80**	0.95**	0.95**	0.91**	0.97**
(14) Adjectives	0.47**	0.57**	0.74**	0.75**	0.71**	0.80**	0.85**	0.79**	0.78**	0.78**	0.86**	0.77**	0.90**	–	0.83**	0.93**	0.93**	0.87**	0.94**
(15) Tools	0.47**	0.43**	0.65**	0.65**	0.58**	0.65**	0.78**	0.71**	0.81**	0.66**	0.75**	0.79**	0.74**	0.73**	–	0.83**	0.83**	0.69**	0.81**
(16) Clothing	0.58**	0.67**	0.85**	0.87**	0.83**	0.87**	0.91**	0.90**	0.85**	0.90**	0.91**	0.82**	0.90**	0.82**	0.74**	–	0.96**	0.88**	0.97**
(17) Toys	0.55**	0.67**	0.86**	0.87**	0.83**	0.87**	0.92**	0.90**	0.87**	0.90**	0.90**	0.86**	0.89**	0.85**	0.80**	0.93**	–	0.87**	0.96**
(18) Other	0.54**	0.69**	0.77**	0.82**	0.84**	0.89**	0.81**	0.84**	0.73**	0.84**	0.82**	0.73**	0.88**	0.80**	0.60**	0.80**	0.81**	–	0.92**
(19) T.S. (Cylex)	0.60**	0.73**	0.89**	0.96**	0.92**	0.94**	0.95**	0.96**	0.90**	0.94**	0.92**	0.87**	0.94**	0.86**	0.75**	0.96**	0.94**	0.88**	–

Intercorrelations between word categories and the total score in a-Cylex (GR), (receptive vocabulary, left diagonally); Intercorrelations between word categories and the total score in a-Cylex (GR), (expressive vocabulary, right diagonally). T.S., Total Score. **p < 0.01.

### Concurrent Validity of the a-CYLEX (GR)

Concurrent validity of the a-CYLEX (GR) was also assessed by examining the association between the standardized values of the a–CYLEX’s (GR) Receptive Vocabulary and the Greek Adaptation of ROWPVT-II in the age group 2:0–3:6 (*n* = 124). As results indicated, a–CYLEX (GR) Receptive Vocabulary was statistically but moderately correlated with ROWPVT-II, [a-CYLEX GR_*receptive Vocabulary*_/ROWPVT – II, *r* = 0.48, *p* < 0.01].

### Frequency of Word Categories for Receptive and Expressive Vocabulary by Age

**FIGURE 1:**
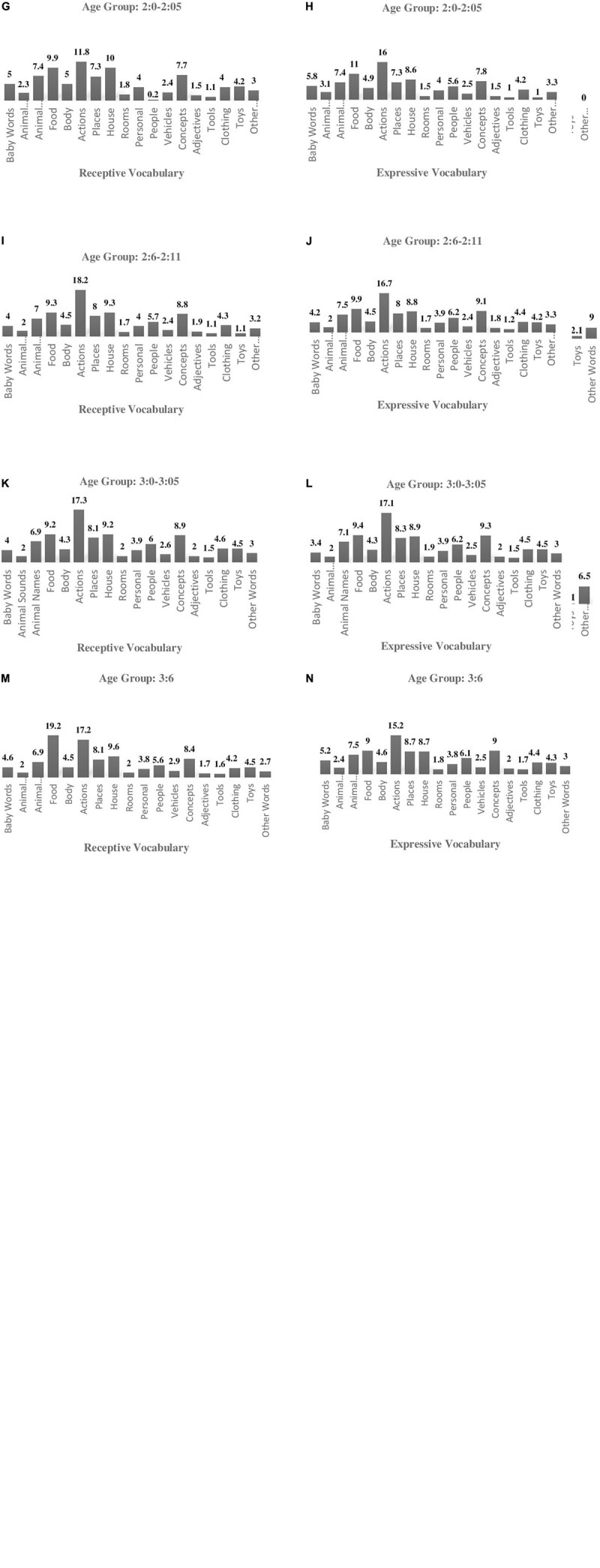
Receptive and expressive vocabulary (word categories) in a-CYLEX (CR) per age group (%). **(A)** Receptive vocabulary per words category (%). **(B,D,F,H,J,L,N)** Expressive vocabulary per word category (%). **(C,E,G,I,K,M)** Receptive vocabulary per word category (%).

The frequency of each word category of the a–CYLEX (GR) for receptive and expressive vocabulary respectively, is presented per age group in Figure 1.

#### Age Group 0:6–0:11

The most frequent word categories in a–CYLEX (GR) regarding receptive vocabulary were actions (28.6%), baby words (19.8%), household objects (8.8%), and food/drink (6.6%) and for expressive vocabulary were baby words (4.4%) and people (0.7%) (see Figures 1A,B).

#### Age Group 1:0–1:5

Similarly, the most prevalent categories for receptive vocabulary were actions (22.8%), baby words (11.6%), household objects (8.8%) and food/drink (7.3%) and for expressive vocabulary were baby words (33.2%), animal sounds (15.6%), people (11.8%) and food/drink (9.3%) (see Figures 1C,D).

#### Age Group 1:6–1:11

The categories with the highest percentages regarding receptive vocabulary were actions (20%), food/drink and household objects (9.1% for each), and concepts (7.3%), and the categories with the highest percentages regarding expressive vocabulary were baby words (17%), food/drink (11.2%), actions (11%), and animal sounds (8.3%) (see Figures 1E,F).

#### Age Group 2:0–2:5

Regarding receptive vocabulary, categories with the highest percentages were actions (11.8%), household objects (10%) food/drink (9.9%) and concepts (7.7%) whereas for expressive vocabulary higher percentages were observed for actions (16%), food/drink (11%) household objects (8.6%) and concepts (7.8%) (see Figures 1G,H).

#### Age Group 2:6–2:11

Regarding receptive vocabulary, categories with the highest percentages were actions (18.2%), food/drink and household objects (9.3% for each), and concepts (8.8%), whereas for expressive vocabulary higher percentages were observed for actions (16.7%), food/drink (9.9%), concepts (9.1%) and household objects (8.8%) (see Figures 1I,J).

#### Age Group 3:0–3:5

Regarding receptive vocabulary, higher percentages were demonstrated in the action’s category (17.3%), the food/drink and household objects categories (9.2% for each), and in the concept category (8.9%). Regarding expressive vocabulary, higher percentages were demonstrated in the action’s category (17.1%), in the food/drink category (9.4%) in the concept category (9.3%) and in the household objects category (8.9%) (see Figures 1K,L).

#### Age Group 3:6

This age group consisted of a small number of participants (*N* = 5). Nevertheless, the frequencies of each word category are presented for comparison purposes. Higher percentages in the receptive vocabulary were observed for the categories of actions (17.2%), household objects (9.6%), food/drink (9.2%), and concepts (8.4%). For the expressive vocabulary, higher percentages were observed for the categories of actions (15.2%), concepts (9%), places and household objects (8.7% for each) (see Figures 1M,N).

### Frequency of Word Categories and Differences in Word Categories by Gender

**FIGURE 2:**
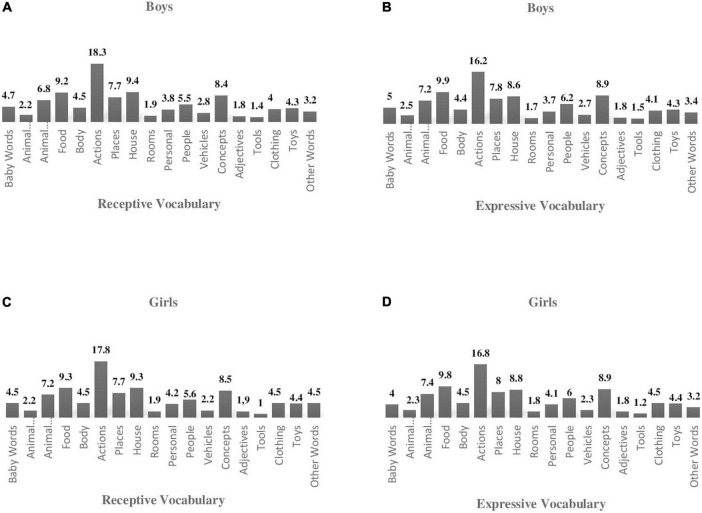
Receptive and experessive volcabulary (word categories) in a-CYLEX (GR) per gender (%). **(A,C)** Receptive vocabulary per words category (%). **(B,D)** Expressive vocabulary per word category (%).

The most prevalent categories regarding receptive vocabulary for boys and girls were actions (boys: 18.3%, girls: 17.8%), household objects (boys: 9.4%, girls: 9.3%), food/drink (boys: 9.2%, girls: 9.3%) and concepts (boys: 8.4%, girls: 8.5%). Additionally, the most prevalent categories observed regarding expressive vocabulary for boys and girls were actions (boys: 16.2%, girls: 16.8%), food/drink (boys: 9.9%, girls: 9.8%), concepts (boys: 8.9%, girls: 8.9%), and animal names (boys: 7.2%, girls: 7.4%). Boys and girls seemed to have similar performance in all the semantic categories (see Figures 2A–D).

Regarding the number of the receptive vocabulary items acquired per word category, the independent-samples “*t*-test” indicated significant gender differences in all categories except for baby words, places, vehicles, concepts and tools (see Table 4) with girls achieving higher scores than boys.

**TABLE 4:** Differences in a–CYLEX (GR) word categories (receptive, expressive vocabulary, and total score) by gender.

Word categories	MGirls (*n* = 105)	*SD*	MBoys (*n* = 89)	*SD*	*t*-value	*df*	*p*
**Receptive vocabulary**							
Baby words	19.10	8.10	17.30	7.47	–1.61	192.00	>0.05
Animal Sounds	9.36	2.78	8.17	3.41	–2.64	169.59	<0.01
Animal Names	30.25	11.31	24.90	14.35	–2.85	166.08	<0.01
Food and Drink	39.31	13.13	33.81	16.82	–2.51	165.06	<0.05
Body parts	19.14	5.48	16.54	7.41	–2.74	159.51	<0.01
Actions	75.21	23.23	66.87	29.40	–2.17	166.38	<0.05
Places	32.31	14.20	28.03	16.16	–1.96	192.00	>0.05
Household objects	38.97	13.25	34.18	16.87	–2.17	165.72	<0.05
Rooms	7.79	3.84	6.87	4.28	–2.30	192.00	<0.05
Personal	17.52	5.89	14.02	7.00	–3.73	172.70	<0.001
People	23.70	10.37	20.08	11.51	–2.28	179.07	<0.05
Vehicle	9.42	4.48	10.06	5.63	0.86	167.00	>0.05
Clothing	18.99	7.99	14.57	9.30	–3.51	174.73	<0.01
Concepts	35.68	16.03	30.85	18.13	–1.97	192.00	>0.05
Adjectives	7.88	3.91	6.56	4.25	–2.24	192.00	<0.05
Toys	18.42	7.73	15.81	9.01	–2.15	174.67	<0.05
Tools	4.69	4.38	5.10	4.17	0.67	192.00	>0.05
Other	13.03	4.43	11.57	5.26	–2.06	172.66	<0.05
**Expressive Vocabulary**							
Baby words	15.77	9.19	14.00	9.16	–1.34	192.00	>0.05
Animal Sounds	8.68	3.22	6.92	3.77	–3.45	174.19	<0.01
Animal Names	28.29	14.03	19.93	16.46	–3.77	173.94	<0.001
Food and Drink	37.31	16.08	27.55	19.54	–3.76	175.51	<0.001
Body parts	17.06	7.56	12.06	9.70	–3.92	167.58	<0.001
Actions	64.00	32.68	44.73	39.77	–3.65	170.33	<0.001
Places	30.40	16.45	21.57	19.48	–3.37	173.10	<0.001
Household objects	33.70	17.59	23.90	17.59	–3.50	172.59	<0.01
Rooms	6.68	4.68	4.82	4.96	–2.68	192.00	<0.01
Personal	15.54	7.07	10.34	8.45	–4.61	172.07	<0.001
People	23.01	11.58	17.08	13.46	–3.26	174.89	<0.01
Vehicle	8.71	5.25	7.48	6.61	–1.42	166.96	>0.05
Clothing	17.30	9.12	11.46	10.51	–4.10	175.66	<0.001
Concepts	33.84	18.60	24.65	21.19	–3.18	176.69	<0.01
Adjectives	6.94	4.38	5.00	4.66	–3.01	192.00	<0.01
Toys	16.69	9.13	11.89	10.64	–3.34	174.63	<0.01
Tools	4.49	4.59	4.21	4.87	–0.40	192.00	>0.05
Other	12.10	4.83	9.31	6.16	–3.47	165.52	<0.01

Regarding the number of expressive vocabulary items acquired, significant gender differences were detected in all categories except for baby words, vehicles and tools (see Table 4). Similar to receptive vocabulary, girls outperformed boys.

### Demographic Variables and Language Development

One-way between subjects ANOVAs were conducted to the whole sample of participants in order to compare the effect of several demographic variables (age, gender, kindergarten attendance, and educational status of parents) on the receptive and expressive vocabulary respectively. The 7th age group (3:6 years old) was excluded from further analysis due to the small number of participants.

### Effect of Age on Receptive/Expressive Vocabulary

**FIGURE 3:**
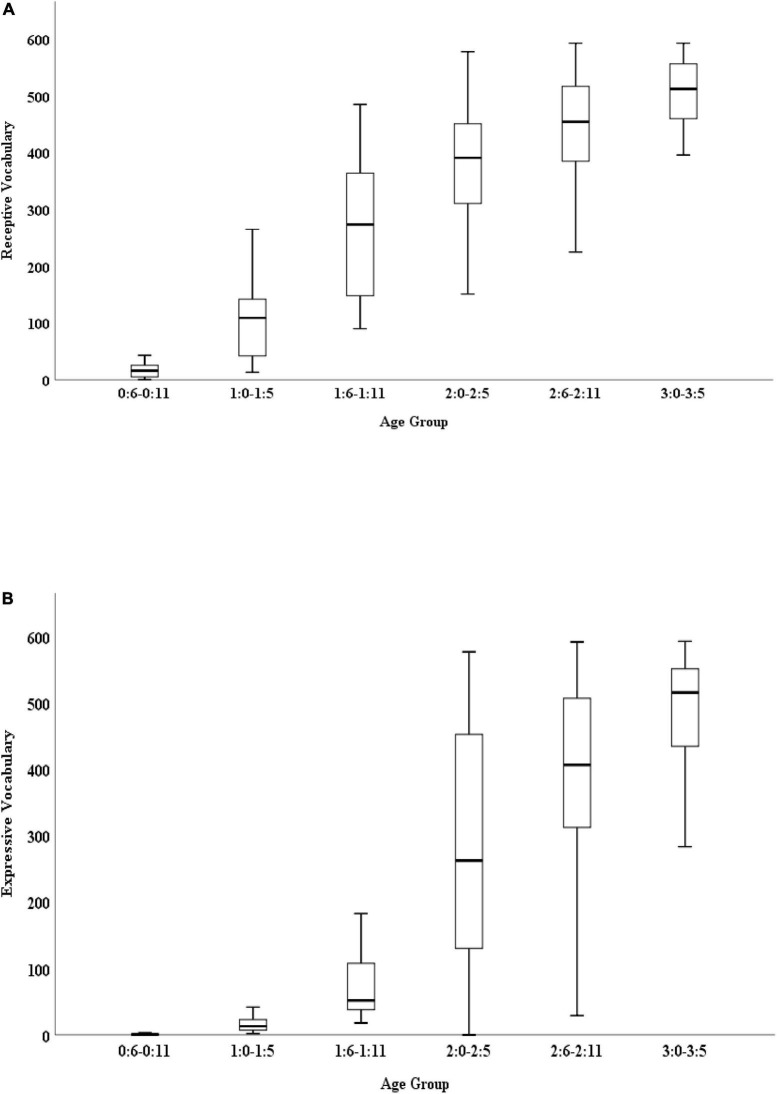
**(A)** Boxplot for receptive vocabulary by age group. **(B)** Boxplot for expressive vocabulary by age group.

There was a significant effect of age group on receptive vocabulary [*F*(5,183) = 77.40, *p* < 0.001] and on expressive vocabulary, [*F*(5,183) = 61.26, *p* < 0.001]. *Post hoc* pairwise comparisons with Bonferroni correction indicated significant mean differences in the receptive vocabulary between the age groups 1:0–1:5 and 1:6–1:11 and between the age groups 1:6–1:11 and 2:0–2:5 (see Table 5). Accordingly, significant mean differences were found for the expressive vocabulary between the age groups 1:6–1:11 and 2:0–2:5, as well as between the age groups 2:6–2:11 and 3:0–3:5 (see Table 6). The variance in each age group is presented in Figures 3A,B, respectively for receptive and expressive vocabulary.

**TABLE 5:** Means (M. O.), standard deviations (SD), range (min–max) and mean differences in the receptive vocabulary between the age groups.

Age groups	M eans (*SD*)	Range_*min–max*_	Mean differences
(a) 0:6–0:11	17.0 (*14.54*)	0.00–43.00	–
(b) 1:0–1:5	118.24 (*95.15*)	13.00–342.00	*M*_*b* – a_ = 101.2*^n.s.^*
(c) 1:6–1:11	278.36 (*124.12*)	9.00–485.00	*M*_*c*– b_ = 160.1*
(d) 2:0–2:5	400.69 (*99.29*)	151.00–578.00	*M*_*d* – c_ = 122.34*
(e) 2:6–2:11	445.70 (*91.44*)	225.00–593.00	*M*_*e* – d_ = 45.00*^n.s^*
(f) 3:0–3:5	492.87 (*88.72*)	106.00–593.00	*M*_*f*– e_ = 47.2*^n.s.^*

*p < 0.05, n.s., not significant.

**TABLE 6:** Means (M. O.), standard deviations (SD), range (min–max) and mean differences in the expressive vocabulary between the age groups.

Age groups	Means (*SD*)	Range_*min–max*_	Mean differences
(a) 0:6–0:11	0.89 (*1.13*)	0.00–3.00	–
(b) 1:0–1:5	17.00 (*15.39*)	2.00–60.00	*M*_*b* – a_ = 16.1*^n.s.^*
(c) 1:6–1:11	81.36 (*74.34*)	18.00–264.00	*M*_*c* – b_ = 64.4*^n.s.^*
(d) 2:0–2:5	305.64 (*165.41*)	5.00–578.00	*M*_*d* – c_ = 224.28*
(e) 2:6–2:11	385.86 (*155.28*)	29.00–593.00	*M*_*e* – d_ = 80.22*^n.s.^*
(f) 3:0–3:5	479.20 (*110.72*)	31.00–594.00	*M*_*f* – e_ = 93.3*

*p < 0.05, n.s., not significant.

### Effect of Gender on Receptive/Expressive Vocabulary

There was a significant effect of gender on receptive vocabulary [*F*(1,187) = 5.50, *p* < 0.05] and on expressive vocabulary, [*F*(1,187) = 12.86, *p* < 0.001) with girls performing better according to parental reports in both receptive (*M*_*receptive*_ = 418.17, *SD* = 144.69) and expressive (*M*_*expressive*_ = 377.81, *SD* = 180.55) vocabulary skills compared to boys (*M*_*receptive*_ = 363.09, *SD* = 178.14 and *M*_*expressive*_ = 273.09, *SD* = 220.90).

### Effect of Kindergarten Attendance on Receptive/Expressive Vocabulary

The effect of kindergarten attendance on receptive and expressive vocabulary was found to be significant for the two conditions (kindergarten attendance vs. no attendance). Significant differences were observed for both receptive vocabulary, [*F*(1,187) = 38.78, *p* < 0.001] and expressive vocabulary, [*F*(1,187) = 44.87, *p* < 0.001). Pairwise mean comparisons indicated significant higher levels of receptive and expressive vocabulary for children who attended kindergarten (*M*_*receptive*_ = 433.43, *SD* = 125.82, *M*_*expressive*_ = 384.40, *SD* = 176.08) compared to children who did not (*M*_*receptive*_ = 281.00, *SD* = 198.76, *M*_*expressive*_ = 179.38, *SD* = 210.08).

### Effect of Educational Status of Parents on Receptive/Expressive Vocabulary

One-way between subjects ANOVAs were conducted to compare the effect of parental educational level on receptive and expressive vocabulary respectively. There was no significant effect of parental educational level on receptive [*F*(5,183) = 0.45, *p* = 0.82] and expressive vocabulary [*F*(5,183) = 0.66, *p* = 0.64] for the four educational categories.

## Discussion

In the present study, the psychometric properties of the a–CYLEX (GR), the frequency of each a-CYLEX (GR) word category as a function of age and gender, gender differences across word categories, and finally the influence of several demographic factors on children’s receptive and expressive vocabulary development were investigated in the geographical region of Crete.

Regarding the reliability of a–CYLEX (GR), a high test-retest reliability was obtained for both receptive and expressive vocabulary in the Cretan-speaking sample, similar to the results presented by Parizi et al. (2013) and by Salagoudi (2019) for regions of Northern Greece. In addition, the high internal consistency of the a–CYLEX (GR) performed in Crete is also supported by previous studies conducted in the Northern part of Greece (Chachoudi, 2012; Salagoudi, 2019). These findings confirm the reliability and validity of the a–CYLEX (GR) (see Table 7).

**TABLE 7:** Summary of results (test–retest reliability and internal consistency) of a-CYLEX and a-CYLEX (GR).

Studies	Instrument	Sample	Region	Test–retest reliability (*r*)		Internal Consistency (Cronbach α)		
				RV	EV	RV	EV	TS
Parizi, 2012; Chachoudi, 2012	a-CYLEX	Typically Developing Children	Thessaloniki	0.997	0.997	–	–	0.966
Salagoudi, 2019	a-CYLEX	Typically Developing Children	Thessaloniki	0.768	0.963	0.936	0.948	–
Helidoni, 2022 (unpublished)	a-CYLEX (GR)	Typically Developing Children	Crete	0.984	0.989	0.96	0.96	0.98

*RV, receptive vocabulary; EV, expressive vocabulary; TS, total score.*

The modest agreement found between the a–CYLEX (GR) Receptive Vocabulary Checklist and the Greek Adaptation of ROWPVT-II does not provide strong support for the concurrent validity of the first. The children’s performance on ROWPVT-II was moderately equivalent to the parents’ observations and reports. Possible reasons for these results might be the different structure of the ROWPVT-II and the a–CYLEX (GR), as well as the different methodological approaches conducted in the two scales for gathering data. CYLEX is a parental report, whereas ROWPVT-II is a formal assessment of the children’s receptive vocabulary administered by the examiner.

Children first learn words that are usually used in their familiar environment. From the onset of vocabulary development, it can be observed that words are recognized from more than one category (Benedict, 1979). In the present study, it was observed that from the beginning of vocabulary development, words from different semantic categories such as baby words, actions, animal sounds, and names, etc. were encountered. In addition, overall vocabulary development generally increased in all semantic categories as a function of age.

A comparison was made between the present study which collected data from children in Crete and Chachoudi’s study (2012) which collected data from children in Northern Greece, concerning the most prevalent semantic categories in receptive and expressive language for each age group. It should be mentioned that in Chachoudi’s study (2012) the categories “baby words” and “animal sounds” were not investigated because when children produce their first real words they can already understand and produce the items in those two categories. Similar results are observed between the two studies regarding the semantic categories most frequently understood and produced by Greek children for each age group. The most prevalent categories for Greek children in a-CYLEX (GR) are actions, house objects, food/drink and concepts categories for both studies. In addition, Salagoudi (2019) found that actions were the most prominent category in the receptive vocabulary from 1:6 to 2:0 years old and the third most prevalent category in the expressive vocabulary from 2:1 to 3:5 years old. It should be mentioned that in the present study, baby words were also a prevalent category from 0:6 to 1:5 years old in both receptive and expressive vocabulary. The results for the tool category were very low for every age group probably because such vocabulary is probably less frequently used by parents.

Furthermore, the variety of linguistic and sociocultural input should be taken into consideration in early word learning (Tardif, 1996). Caselli et al. (1995) based on parental reports compared the receptive and expressive lexical development of 659 English infants and 195 Italian infants between 8 and 16 months and found that in both languages, nouns are acquired first and verbs emerge when children have acquired at least 100 words. In the Caselli et al. (1995) study verbs were reported earlier for receptive vocabulary than for expressive vocabulary. Benedict (1979), reported that the two dominant categories in children’s early vocabularies were nominals and action words and that action words were more evident in the children’s early comprehension vocabulary than in the production vocabulary as also reported by Caselli et al. (1995). The previous findings regarding the earlier emergence of action words in receptive vocabulary are also consistent with the findings of the present study regarding the similar age groups. More specifically for the age group 1:0 – 1:5 for the receptive vocabulary the actions percentage was (22.8%) whereas for the expressive vocabulary the corresponding percentage was (2.4%) and for the age group 1:6 – 1.11 for the receptive vocabulary the actions percentage was (20%) whereas for the expressive vocabulary the corresponding percentage was (11%). Actions were more evident in the children’s early receptive vocabulary in comparison to the expressive vocabulary. It should be taken into consideration that from the 18 semantic categories contained in the a-CYLEX (GR) checklist, only one category was represented by actions. Hence, one must not conclude that Greek-learning children acquire more verbs than nouns in their early language development.

In addition, the results suggested a significant lexical growth as a function of age across the receptive and the expressive domains, specifically, from 1:6 to 2:5 years for receptive vocabulary and from 2:0–2:5 and 3:0–3:5 years for expressive vocabulary. These results agree with the results of Chachoudi (2012) who found that children in the Northern part of Greece showed a vocabulary growth in the receptive vocabulary from 1:6 to 2:0 and a vocabulary spurt in the expressive vocabulary from 2:0 to 2:6 years old. Karousou and Petrogiannis (2014) found that the most significant changes in receptive vocabulary took place from 1:1 to 1:2 and the most significant changes in expressive vocabulary took place from 1:3 to 2:2 years of age. D’Odorico et al. (2001) reported that vocabulary spurt can take place at different ages. Papaeliou and Rescorla (2011) investigated the vocabulary development in three age groups (1:6–1:11, 2:0–2:5, 2:6–2:11) using the Greek adaptation of Rescorla’s Language Development Survey between Greek children and US children, and found that the acquisition of vocabulary was rather slower in Greek-speaking children at the first age group (1:6–1:11) in comparison to English-speaking children. This finding is probably related to the complex morphology and structure of the Greek Language (Papaeliou and Rescorla, 2011).

With regards to gender, statistically significant differences were found in receptive and expressive vocabulary with girls outperforming boys in both. This was supported by Parizi (2012) who also found that girls outperformed boys in a–CYLEX (GR) in both receptive and expressive vocabulary. Papaeliou et al. (2003) investigated the vocabulary in 300 toddlers using the Greek Adaptation of the Developmental Language Survey and found that girls used more vocabulary in comparison to boys. The findings of the present study regarding receptive and expressive vocabulary and gender are also in agreement with the findings of Umek et al. (2008), who investigated the effect of children’s gender on toddler language development in 953 Slovenian toddlers aged 16–30 months and found that girls were more linguistically competent in comparison to boys. This is corroborated by other existing studies (Bornstein and Haynes, 1998; Rescorla and Alley, 2001; Bauer et al., 2002; Fenson et al., 2008). Similar findings were noted by Stolt et al. (2008) who found a significant effect between gender and expressive vocabulary as early as 1:3 and 1:6 years old for girls.

It is usually expected that children whose parents have a higher educational level will present better receptive and expressive vocabulary. Parizi (2012) who investigated receptive and expressive vocabulary in 186 children using the a-CYLEX found that parents’ higher educational level had a significant effect on children’s expressive vocabulary. Moreover, Umek et al. (2008) who investigated the effect of parental education on toddler language development aged 16–30 months found that parental education had a significant but small effect on the children’s language competence, with toddlers whose parents had a higher educational level showing a larger vocabulary and using longer and more complex utterances. Interestingly, Feldman et al. (2000) administered certain properties of the MacArthur-Bates CDI to a large sample of 2156 children aged 1 and 2 years old and found that children whose mothers had a lower educational status presented higher scores in words understood and produced in comparison to children whose mothers had a higher educational status. Similarly, Bavin et al. (2008) administered the MacArthur-Bates CDI to a large sample of 1447 Australian children aged 8 months, 1 and 2 years and also found that 1 year old children from the lower socioeconomic areas understood more words from children at the same age from the higher socioeconomic areas. Parental education in the present study did not seem to have a significant effect on the children’s receptive and expressive vocabulary as also found by Salagoudi (2019). It should be noted that in the present study there was a ceiling effect in levels of parental education because the majority of the parents had higher than high school education, preventing the drawing of conclusions regarding parental education and vocabulary development. In addition, the investigators hypothesize that a possible reason for these differences encountered regarding parental education and vocabulary development might be that parents with a lower educational level might overestimate their children’s language abilities, whereas parents with a higher educational level might make more cautious estimates.

Furthermore, a significant effect was found between receptive and expressive vocabulary and kindergarten attendance. This is in agreement with the results of Parizi (2012). In addition, the results are in accordance with Hansen and Hawkes (2009) findings that children’s formal group care attendance was positively associated with expressive vocabulary. In kindergarten, children receive considerable stimuli in the classes through songs, play and interaction with each other and with the teachers, thus presenting them with opportunities to learn more vocabulary.

The a-CYLEX (GR) is a checklist based on parental reports. It is important to mention that although parental reports are based on the parents’ observations, the method of using parental recordings is more representative of the child’s progress at this age given that parents are in daily contact with their child in a variety of situations, unlike laboratory conditions where the child might be inhibited by shyness or mood during the recording of specific observations (Fenson et al., 2008). On the other hand, parents may sometimes underestimate or overestimate their child’s vocabulary skills (Roberts et al., 1999).

The limitations of the present study are that each age group is not represented equally by the same number of children. Therefore, these results cannot be generalized to the population yet. In a future study, each age group could be represented by an equal number of children. In addition, the majority of the children who participated in this study resided in urban areas. In a future study, a larger proportion who live in rural areas should participate in order to compare the receptive and expressive vocabulary skills of children who live in different areas as investigated in the study of Tzouriadou and Manavopoulos (2007). It is also recommended, in the process of standardization of a–CYLEX (GR), to administer it to a group of children with speech and language disorders as performed in the Oktapoti et al. (2016) study, in order to compare their receptive and expressive vocabulary profile with that of children with normal language development, thereby deriving indices of clinical validity of the instrument.

The current study validated certain psychometric properties of the a–CYLEX (GR) for the region of Crete. Findings obtained regarding vocabulary acquisition and vocabulary composition in Greek children who speak a Southern dialect supported the findings from previous work (Chachoudi, 2012; Parizi, 2012; Parizi et al., 2013; Salagoudi, 2019) in Northern Greece. The a–CYLEX (GR) could also give a better insight to parents and specialists regarding the development of various word categories in children. Overall, the a–CYLEX (GR) could be considered as an efficient screening tool for assessing receptive and expressive vocabulary and according to its outcome, children can be referred for further evaluation to specialists and if needed for early intervention.

## Data Availability Statement

The raw data supporting the conclusions of this article will be made available by the authors, without undue reservation.

## Ethics Statement

The studies involving human participants were reviewed and approved by Ethics committee of the University of Macedonia, Department of Educational and Social Policy. Written informed consent to participate in this study was provided by the participants’ legal guardian/next of kin.

## Author Contributions

MH gathered the data in Heraklion, Crete, Greece. All authors provided valuable advice regarding the analysis of the data of the present study and made significant contributions to the statistical analyses and writing of the manuscript.

## Conflict of Interest

The authors declare that the research was conducted in the absence of any commercial or financial relationships that could be construed as a potential conflict of interest.

## Publisher’s Note

All claims expressed in this article are solely those of the authors and do not necessarily represent those of their affiliated organizations, or those of the publisher, the editors and the reviewers. Any product that may be evaluated in this article, or claim that may be made by its manufacturer, is not guaranteed or endorsed by the publisher.

## Appendix

**Table:** Test–retest reliability in the R.V (receptive vocabulary) and E.V. (Expressive Vocabulary) per age.

Age	R.V. test	R.V. retest	E.V. test	E.V. Retest	Age	R.V test	R.V. retest	E.V. test	E.V. retest
0:06:07	7	13	0	0	2:04:10	395	396	335	341
0:06:18	21	23	0	1	2:04:28	572	575	531	540
0:07:02	0	0	1	1	2:05:00	561	563	560	563
0:08:16	3	29	1	4	2:06:12	356	448	124	179
0:10:09	30	18	3	4	2:06:28	336	401	30	17
0:10:18	13	56	2	12	2:07:14	374	288	142	116
0:11:14	12	11	0	1	2:07:10	367	387	264	284
0:11:16	74	88	15	21	2:09:03	503	519	468	482
1:01:03	25	38	7	13	2:09:16	457	459	423	429
1:01:10	126	173	25	36	2:11:24	535	583	436	510
1:01:12	124	141	16	12	3:00:05	530	530	528	529
1:01:18	109	174	3	1	3:01:09	489	555	457	502
1:03:07	113	133	3	3	3:01:10	588	576	577	566
1:03:14	175	191	42	49	3:01:15	511	504	514	510
1:04:07	265	288	22	35	3:01:19	421	517	389	457
1:04:18	142	179	23	31	3:01:27	522	547	547	583
1:04:20	79	83	10	7	3:02:21	427	414	465	416
1:07:22	250	250	47	46	3:03:00	567	587	569	585
1:08:20	134	174	45	101	3:03:00	528	539	539	556
1:10:01	356	375	28	60	3:03:13	496	557	524	564
1:10:20	415	452	176	213	3:04:18	406	478	393	482
1:11:22	301	344	109	147	3:04:22	447	515	435	409
1:11:28	333	371	115	169	3:04:25	550	551	545	499
2:00:09	391	404	138	158	3:05:02	174	156	394	438
2:00:12	510	563	441	549	3:05:12	553	581	554	585
2:00:18	527	556	527	556	3:05:15	367	365	228	290
2:00:23	390	424	132	181	3:06:02	347	336	341	447
2:01:02	387	429	344	409	3:06:04	520	508	450	449
2:01:18	299	392	299	393					
2:02:15	450	507	290	317					
2:02:16	503	516	5	7					

*Mean.(S.D)^R.L.test^ = 329.76(189.34), Mean.(S.D)^R.L.retest^ = 353.05(193.87), Mean. (S.D.)^E.V.test^ = 248.03(217.92), Mean.(S.D.)^E .V.retest^ = 268.90(225.23).*
